# RELIABILITY AND VALIDITY OF A REDUCED SET OF NAVIGATION ITEMS IN COMMUNITY-DWELLING OLDER ADULTS

**DOI:** 10.1093/geroni/igac059.2950

**Published:** 2022-12-20

**Authors:** Michael Prevratil, Dorota Kossowska-Kuhn, Nicholas Gray, Neil Charness

**Affiliations:** Florida State University, Tallahassee, Florida, United States; Florida State University, Tallahassee, Florida, United States; Florida State University, Tallahassee, Florida, United States; Florida State University, Tallahassee, Florida, United States

## Abstract

Navigation is a complex skill that is used in everyday living, whether it be to travel across a country or to travel to a local store. How one successfully navigates through their environment involves many different processes, including spatial navigation, route generation, and orientation. An issue with investigating those separate constructs within navigation is the number of questions required to assess them reliably. As a part of a larger project, a large sample of community-dwelling older adults (ages 60–90) completed an online survey answering questions related to navigation. Among those were three subscales: a general wayfinding subscale, a subscale asking how often they felt lost when moving around near and far spaces, and a subscale asking how often they needed help navigating around near and far spaces. Each of these subscales contained fewer than eight items. The goal of the analysis was to determine the reliability and validity of these subscales, and this was accomplished through calculating Cronbach’s α and an exploratory factor analysis (EFA). Cronbach’s α for each of the individual subscales were above 0.8, indicating high reliability. EFA results output five unique factors. Noticeably, the wayfinding subscale was broken into two factors, one for route generation ability and one for mental mapping ability. while the “Feeling Lost” and “Needing Help” subscales produced a dichotomy between nearer distances (ex. Your immediate neighborhood) and farther distances (ex. Your state). This contrast, along with its implications, are discussed further.

